# Using Naïve Bayesian Analysis to Determine Imaging Characteristics of KRAS Mutations in Metastatic Colon Cancer

**DOI:** 10.3390/diagnostics7030050

**Published:** 2017-09-02

**Authors:** Yash Pershad, Siddharth Govindan, Amy K. Hara, Mitesh J. Borad, Tanios Bekaii-Saab, Alex Wallace, Hassan Albadawi, Rahmi Oklu

**Affiliations:** 1 Department of Radiology, Division of Vascular & Interventional Radiology, Mayo Clinic, Phoenix, AZ 85054, USA; pershad.yash@mayo.edu (Y.P.); albadawi.hassan@mayo.edu (H.A.); 2 Department of Radiology, Massachusetts General Hospital, Boston, MA 02114, USA; sgovindan@partners.org; 3 Department of Radiology, Mayo Clinic, Phoenix, AZ 85054, USA; hara.amy@mayo.edu (A.K.H.); wallace.alex@mayo.edu (A.W.); 4 Department of Hematology/Oncology, Mayo Clinic, Scottsdale, AZ 85259, USA; borad.mitesh@mayo.edu (M.J.B.); bekaii-saab.tanios@mayo.edu (T.B.-S.)

**Keywords:** naïve Bayesian classification, radiogenomics, RAS mutation, machine learning, natural language processing

## Abstract

Genotype, particularly Ras status, greatly affects prognosis and treatment of liver metastasis in colon cancer patients. This pilot aimed to apply word frequency analysis and a naive Bayes classifier on radiology reports to extract distinguishing imaging descriptors of wild-type colon cancer patients and those with v-Ki-ras2 Kirsten rat sarcoma viral oncogene homolog (KRAS) mutations. In this institutional-review-board-approved study, we compiled a SNaPshot mutation analysis dataset from 457 colon adenocarcinoma patients. From this cohort of patients, we analyzed radiology reports of 299 patients (> 32,000 reports) who either were wild-type (147 patients) or had a KRAS (152 patients) mutation. Our algorithm determined word frequency within the wild-type and mutant radiology reports and used a naive Bayes classifier to determine the probability of a given word belonging to either group. The classifier determined that words with a greater than 50% chance of being in the KRAS mutation group and which had the highest absolute probability difference compared to the wild-type group included: “several”, “innumerable”, “confluent”, and “numerous” (*p* < 0.01). In contrast, words with a greater than 50% chance of being in the wild type group and with the highest absolute probability difference included: “few”, “discrete”, and “[no] recurrent” (*p* = 0.03). Words used in radiology reports, which have direct implications on disease course, tumor burden, and therapy, appear with differing frequency in patients with KRAS mutations versus wild-type colon adenocarcinoma. Moreover, likely characteristic imaging traits of mutant tumors make probabilistic word analysis useful in identifying unique characteristics and disease course, with applications ranging from radiology and pathology reports to clinical notes.

## 1. Introduction

Radio-genomics (RG), which correlates cancer imaging findings and genetic alterations, is an emerging science with important implications for cancer treatment [1,2]. An alternative to direct image-outcome analysis, RG allows identification of reasons for these associations and enables access to much larger genomic databases compared to smaller imaging datasets for predicting outcomes [3]. RG provides a method for extracting more information from standard imaging results, and once a high level of accuracy can be achieved, could help avoid or reduce the use of more specialized and costly genetic profiling [4]. Predictive keywords derived from imaging reports and based upon the radio-phenotype can potentially diagnose genetic variants of a cancer, thereby expediting and focusing cancer treatments in the era of gene-based therapeutics. Previous applications of RG include breast cancer, liver cancer, and glioblastoma [2,3,4,5].

Historically, the determination of imaging differences of different cancer variants has been based upon manual review of images from large cohorts of patients [5,6,7]. In this research, we explore the possibility of using natural language processing of radiology reports in patients with colon cancer and a naïve Bayes classification algorithm to determine which predictive key terms from the imaging reports are likely for a given genetic variant of colon cancer.

Particularly, this research applied the Bayesian classifier to colorectal cancer (CRC), as it ranks second in cancer-related deaths in the U.S. and genotype greatly affects patient prognosis [8]. Since glandular cells play a key role in mucus secretion in the colon, a majority of CRC patients suffer from epithelial adenocarcinoma, which affects glandular cells from the gradual accumulation of genetic mutations and epigenetic alterations [9,10]. 

Key genetic alterations in CRC patients with prognostic value involve a mutant v-Ki-ras2 Kirsten rat sarcoma viral oncogene homolog (KRAS), the most common RAS mutation, which between 30% and 50% of CRC patients express [11,12]. KRAS is a proto-oncogene downstream from the epidermal growth factor receptor (EGFR) that has predicted poorer outcomes, since KRAS mutations create resistance to anti-EGFR antibodies [11,12,13]. Thus, identifying the genotype of CRC patients, particularly KRAS mutants, has become increasingly important for prognosis and treatment strategies [14].

Since identification of the genotype of patients is at its core a classification problem, classification via machine learning, a process by which computers can extract patterns in data and make decisions based upon this new knowledge, is a well-suited solution [15,16]. Naïve Bayes classifiers are simple but flexible probabilistic classifiers that can be trained via machine learning [16]. They have a wide range of applications including spam filtering, self-driving cars, social media analysis, and financial modeling [17]. For example, in spam-filtering, a naïve Bayes classifier computes the probability of a test input (email) being class *S* (spam) based upon (given) the presence of *W* (predictor word). Mathematically, the spam classification algorithm is expressed as

(1) $$P\left(S│W\right)=\;\;\frac{\left(P\left(W│S\right)\;\times \;P\left(S\right)\right)}{(P\left(W│S\right)\;\times \;P\left(S\right)+P\left(W│H\right)\;\times \;P\left(H\right)}$$

where *P(S|W)* is the probability that a message is spam knowing a specific word is present, *P(S)* is the probability that any given message is spam, *P(W|S)* is the probability of the word appearing in all spam messages, *P(H)* is the probability that any given message is not spam (is “ham”), and *P(W|H)* is the probability that the word appears in ham messages.

Much like how a naïve Bayesian algorithm can classify an email as spam or not-spam, the algorithm can also classify a radiology report as belonging to a genetic variant or wild-type of a given cancer. Particular words, potentially those associated with imaging characteristics written up in the report, have different probabilities of occurring in free-text radiology reports of genetic variants versus wild-type cohorts. The difference in the probabilities between the different report cohorts is the feature which naïve Bayes uses to ultimately calculate the probability that a certain report comes from a certain cohort.

This pilot study applied word frequency analysis and a naive Bayes classifier on free-text radiology reports to extract distinguishing imaging descriptors of wild-type colon cancer patients and those with KRAS mutations.

## 2. Materials and Methods 

### 2.1. Patient Acquisition and Parsing Radiological Reports

In this institutional-review-board-approved study (2010P000510, 13 April 2010; MGH IRB), SNaPshot mutation analysis identified the genotype of 457 colon adenocarcinoma patients [14]. Of these, our study focused on 299 with a clear genetic SNaPshot of either KRAS or wild-type, of which approximately half had wild-type and half had KRAS mutations.

With the labeled data, the program then filtered the dataset and removed reports with no clinical significance (e.g., billing documents) to isolate only relevant reports. With the relevant, labeled reports in the processing pipeline, we tokenized the text within these reports into individual words, stemmed words to eliminate variability of conjugations, and used a simple negative word proximity check to tag words with a negative context if applicable, as in previous studies [18,19]. Finally, the program iterated through the reports, adding new words it encountered to the database and incrementing respective counts for repeats.

### 2.2. Naïve Bayesian Classification

Once the program provided the output of the distinct words and the number of appearances of each word in reports of KRAS and wild-type patients respectively, the Bayesian classifier, using the equation described above, computed the probability a report including that particular word comes from a patient with a KRAS or wild-type genotype. This probability value is a measure of the word’s presence in the report as a predictor of a CRC patient’s genotype. 

### 2.3. Statistical Analysis

With the frequency and probability values for each word in KRAS and wild-type reports, we noted the words with the highest absolute difference in frequency and probability between KRAS and wild-type. Finally, for the key words which were associated with either KRAS or wild-type, a Wilcox signed rank test evaluated the presence of all of the words as a predictor of genotype.

## 3. Results

**Figure 1:**
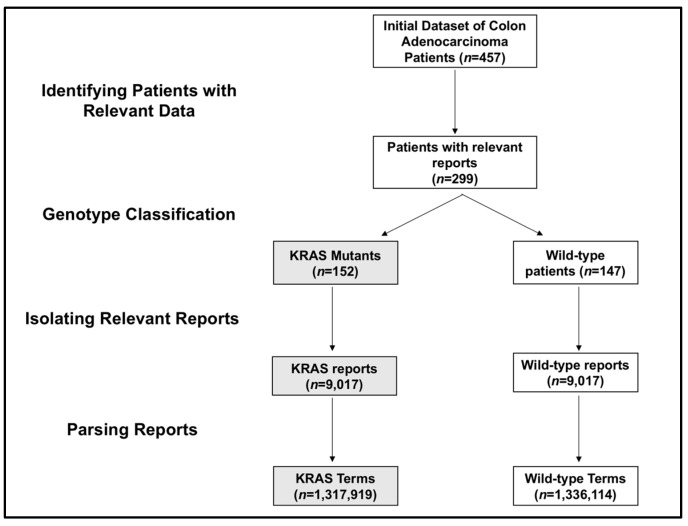
Flowchart of patient selection and data mining. The process of identifying relevant patients and reports to generate KRAS and wild-type terms is shown. KRAS patients, reports, and terms are shown in grey, while wild-type patients, reports, and terms are shown in white.

This pilot study analyzed a total of 299 patients, who either were the wild-type (147 patients) or had a KRAS (152 patients) mutation. For this population, we acquired 18,046 reports from electronic health records. The KRAS mutation group included 9017 reports, and the wild-type group included 9029 reports. Breaking up the reports into word and phrase tokens created 1,317,919 distinct terms for KRAS patients and 1,336,114 distinct terms for wild-type patients (Figure 1).

By dividing the number of KRAS or wild-type reports the words appeared in by the total number of KRAS or wild-type reports, we obtained the frequencies of the words in KRAS and wild-type reports. “Metastas (es)” had the highest frequency in both types of reports and the lowest percent difference between the reports of 19%. The phrase “[no] discrete”, which appeared in higher frequency in wild-type reports, had the highest percent difference between the two types of reports at 72%. “Hypoattenuat(ing)” and “innumerable” had the highest percent difference in frequencies for KRAS predictive terms at 51%.

Next, the Bayesian probability of a KRAS or wild-type patient’s radiology report including the term is computed. Words with a greater than 50% chance (range 55–63%) of being in the KRAS mutation group and which had the highest absolute probability difference compared to the wild-type group included: “numerous”, “several”, “innumerable”, “confluent”, “metastas (es)”, and “hypoattenuat(ing)”. Conversely, words with a greater than 50% chance (range 60–66%) of being in the wild type group and with the highest absolute probability difference included: “[no] abnormal”, “[no] recurrent”, “few”, and “[no] discrete”.

**Figure 2:**
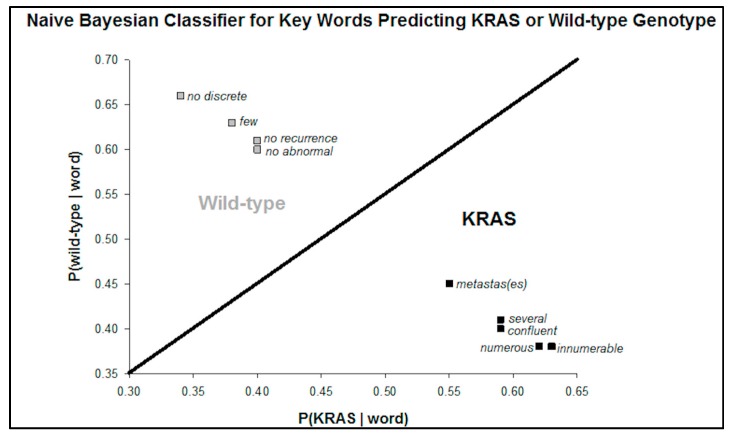
This scatterplot of the probabilities of KRAS or wild-type for predictive terms. The probabilities represent the chance that a report arises from a KRAS or wild-type patient given the term is present. The terms are clustered into KRAS predictors and wild-type predictors.

A scatterplot of the probability of each genotype given the presence of a word in the report demonstrates two clear classifications of these key words (Figure 2). The phrase “no discrete” had the highest percent difference between the probability of predicting a wild-type versus KRAS genotype.

**Figure 3:**
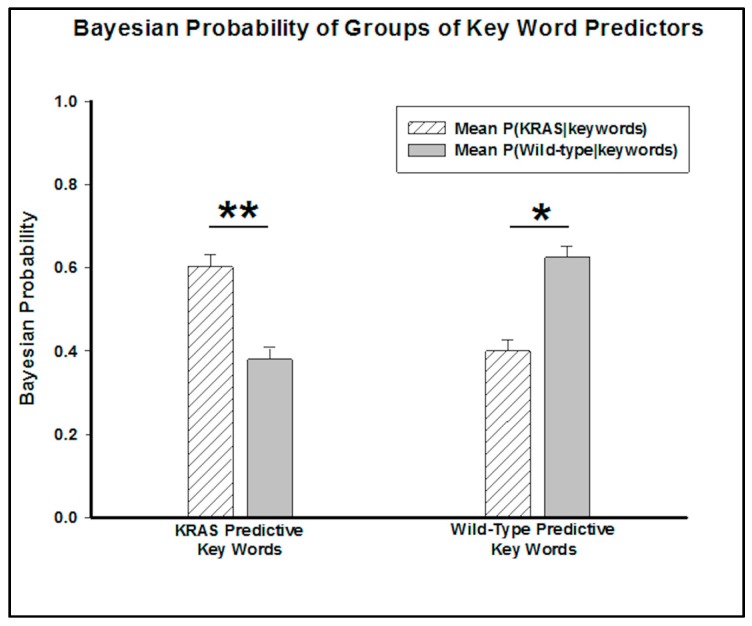
Bar graph comparing the mean KRAS and wild-type probabilities for the groups of terms. The mean probabilities of a patient having a KRAS or wild-type genotype if the predictive key words are in their reports are shown. These groups of key words are statistically significant as key words (** = *p*-value < 0.01, * = *p*-value < 0.05).

By grouping these words into predictive key words for KRAS mutants and wild-type genotypes, a Wilcox signed rank test determined the predictive capability of the groups. The *p*-value (*p* = 0.005) associated with the combination of KRAS predictors suggests that a patient whose report includes all of these words has less than a 1% chance of not having a KRAS mutation. Conversely, the *p*-value (*p* = 0.03) of the key words appearing in wild-type radiology reports suggests that the patient has less than a 5% chance of not having a wild-type genotype (Figure 3).

## 4. Discussion

The purpose of this study was to use a naïve Bayesian classifier to identify predictive keywords from imaging characteristics written up in radiology reports. Reliably identifying the genotype from reports from the medical imaging already performed has the potential to eliminate extraneous genotype tests and direct genotype work-ups.

After parsing the free-text radiological reports and associating them with the appropriate genotype, the program calculated the frequency for each distinct word in reports of a KRAS or wild-type genotype patient. From these frequencies, the Bayesian classifier computed the probability of a patient having a genotype based upon the presence of a predictor key word in their reports.

The key words associated with a higher probability of arising from a report of a KRAS patient (*p* < 0.01) clearly indicated more aggressive adenocarcinoma, with words like “numerous”, “several”, “innumerable”, “confluent”, “metastas(es)”, and “hypoattenuat(ing)”. Conversely, the key phrases, such as “no recurrence”, “no abnormal”, “few”, “no discrete”, associated with the wild-type genotype indicated less aggressive presentations. Such results corroborate studies associating KRAS mutations with cancer that presents more aggressively and associate the genotype with poorer prognosis [15,17,18,19]. 

The application of radiogenomics to colon adenocarcinoma has great clinical significance due to the effect of genotype on treatment options. Since KRAS is downstream from EGFR, KRAS mutations result in resistance to EGFR antibodies. The certainty of the prediction of the genotype of patients in this study can provide clinicians with an important link between imaging and genotype to guide their treatments without having to perform genome sequencing.

While these results identify specific, clinically relevant key words, this pilot study does have limitations. First, the Bayesian classifier only identified these predictor key words from the training set, but we have not yet implemented a method for determining the likelihood that a given report belongs to either a KRAS or wildtype patient based on the words within the report. Such predictive capabilities are a promising future direction, but would require larger training data sets.

Moreover, the study took the approach of analyzing radiological reports rather than the images themselves and therefore is limited by the variability in which the same findings can be expressed in a radiology report. Another limitation of using text from reports is that, while this study analyzed reports from one institution, radiologists from different backgrounds or regions may use different descriptors for the same imaging features. Both standardizing feature extraction from imaging and then performing this analysis and applying the algorithms and key terms extracted from one patient cohort to another distinct one are promising future directions that could potentially lead to more robust results than could be obtained in our pilot study.

For colon cancer, surgical resection is the most common treatment [20], so pathology reports also contain clinically relevant search terms that can distinguish KRAS mutants and wild-type genotypes. The combination of pathological and radiological reports is a future direction to potentially increase accuracy of RAS status predictions. Finally, this probabilistic approach to analyzing text in order to extract predictive features has the potential to be applied to a variety of data sources from clinical and hospital notes to radiology and pathology notes for a variety of diseases.

This pilot study successfully parsed free-text radiological reports and applied a naïve Bayesian classifier to find predictive key words to identify genotypes. This process can be applied to a variety of other diseases other than colon adenocarcinoma. Despite its limitations, it represents a unique approach to doing an initial highly automated review of radiology reports and extracting the associated imaging features which correlate to genotypic variations.

## 5. Conclusions

The purpose of this pilot study was to analyze the frequency of words and use a naïve Bayesian classifier to identify imaging predictors of genotype from radiology reports. The classification algorithm determined that the words that occurred with a higher frequency in KRAS reports, and thus were predictors of the KRAS mutation, were associated with more aggressive imaging features. In contrast, wild-type key words suggested a less aggressive radiophenotype for colon adenocarcinoma patients. For colon adenocarcinoma, extracting genotype from imaging data is extremely promising, as the presence of a KRAS mutation greatly alters the treatment plan for patients. The certainty of the prediction of the genotype of patients in this study can provide clinicians an important link between imaging and genotype to guide their treatments without having to perform genome sequencing.

More generally, these results demonstrate that characteristic imaging traits exist which can reveal the genetic mutations of all cancerous tumors. Probabilistic analysis of radiological reports, or even other clinical notes, has the potential to identify disease course associated with mutated oncogenes and presents a quicker, cheaper alternative to genome sequencing.
